# L5 Fracture Dislocation Secondary to Cold Abscess Treated by Posterior Corpectomy With Expandable Cage Placement

**DOI:** 10.7759/cureus.8756

**Published:** 2020-06-22

**Authors:** Joseph R McFarland, Daniel Branch, Adam Gonzalez, Gerald Campbell, Rishi R Lall

**Affiliations:** 1 Radiology, University of Texas Medical Branch at Galveston, Galveston, USA; 2 Neurosurgery, University of Texas Medical Branch at Galveston, Galveston, USA; 3 Pathology, University of Texas Medical Branch at Galveston, Galveston, USA

**Keywords:** spine, lumbar, corpectomy

## Abstract

Infections of the lumbar spine can have serious sequelae, including neurological deficits, paralysis, and death. Prolonged infection can result in fracture of the vertebrae, local abscesses, and infiltration and compression of local vascular structures. In cases with significant instability or neurological compromise, a common treatment approach is vertebral corpectomy with interbody cage followed by long-term antibiotics.

The following case describes a patient with a three-month history of progressively worsening lower back pain, lower extremity radiculopathy, and bilateral lower extremity edema, in the setting of a nontraumatic three-column fracture dislocation of L5 with grade 4 retrolisthesis of L4 on L5. A posterior-only corpectomy with placement of an expandable cage, to be followed by pedicle screw placement from L3-S1/ilium, was performed. The procedure was successful, and the patient was discharged on postoperative day 5 without complication and with resolution of his edema. Histopathological analysis demonstrated acute and chronic inflammation, but extensive tests and cultures failed to identify a causative organism.

This case highlights several interesting features, including a technically challenging and seldom-performed procedure, as well as the ability of lumbar spinal infections to present with leg edema due to involvement the inferior vena cava and iliac vessels. For patients with three-column fractures of L5 due to an inflammatory process or trauma, a single-stage posterior corpectomy with placement of an expandable cage may be considered as an appropriate treatment option.

## Introduction

Approximately 26,000-65,000 people are affected by vertebral osteomyelitis annually, and 30%-70% present with no signs of prior infection [1]. Epidural abscess development is a known complication of vertebral osteomyelitis in as many as 10%-20% of cases [2]. The mortality rate of spinal infections may be as high as 20%, stressing the importance of early recognition and intervention [3]. For those who survive pyogenic vertebral osteomyelitis, approximately 16% of patients have been shown to suffer from residual neurological deficits, while over 30% of patients will have persistent back pain after recovery [4].

Vertebral infectious osteomyelitis can result in vertebral fracture, and these fractures can be further complicated by spondylolisthesis [5]. Complications in the lumbar spine result from local invasion and mass effect and include paravertebral abscesses, psoas abscesses, and compression of vasculature, such as the inferior vena cava (IVC) and iliac veins, which presents as lower extremity edema [6,7]. The etiologies of pyogenic spinal infections are numerous and include Staphylococcus aureus (most common and typically epidural), Enterococcus species, Escherichia coli, Streptococcus pneumoniae, Salmonella species, Klebsiella species, and Pseudomonas aeruginosa [3,8]. Granulomatous etiologies include Brucella species, Mycobacterium tuberculosis, various fungi, and parasites [3,8]. Unfortunately, the microorganism is not identified in up to one-third of cases despite extensive diagnostic evaluation [3].

Cold abscesses refer to pyogenic collections that form in the absence of inflammatory symptoms or pain [9,10]. Cold abscesses in the context of the spine are almost exclusively in reference to tuberculosis in literature, but other potential etiologies include Brucella, pyogenic spondylitis with preexisting hyper-IgE syndrome, metastasis, multiple myeloma, and soft tumors [9,10].

The Infectious Disease Society of America 2015 guidelines recommend immediate surgical intervention and empiric antimicrobials for native vertebral infections with neurological compromise. Surgical intervention is also warranted in cases of intractable pain, unstable deformity, and refractory disease [11]. Vertebral osteomyelitis has been shown to present as spinal compression or burst fractures [5]. Lumbar burst fractures can be treated via corpectomy and fusion of the adjacent levels [12]. This procedure allows for adequate stabilization and decompression, as well as maintenance of vertebral height [12]. For unstable lumbar burst fractures, a corpectomy with cage placement can be approached in various ways, including anteriorly, posteriorly, laterally, and combined [13]. Currently, there is no consensus on the optimal approach for this procedure, and the surgeon must weigh many factors, including the neurological deficits, anatomical variations, and deformity degree [13]. Anterior and lateral approaches are generally preferred due to better exposure and less retraction of the spinal cord and surrounding structures, resulting in less risk of neurological deficits [14]. In certain cases, however, anterior approaches may be anatomically unfeasible or contraindicated due to abdominal anatomy or risk of vascular injury. Posterior-only L5 corpectomy is a challenging procedure but may offer the potential of less morbidities, faster operating time, and faster rehabilitation in comparison to the anterior or combined approaches [15-18].

The following case describes a patient who was found to have a cold abscess resulting in a three-column fracture dislocation of L5 with grade 4 retrolisthesis of L4 on L5. He was treated surgically with posterior-only L5 corpectomy with expandable cage placement due to high risk of vascular injury with an anterior approach. We provide a technical description of this seldom-performed procedure and potential indications for use of a posterior-only approach.

## Case presentation

**Figure 1:**
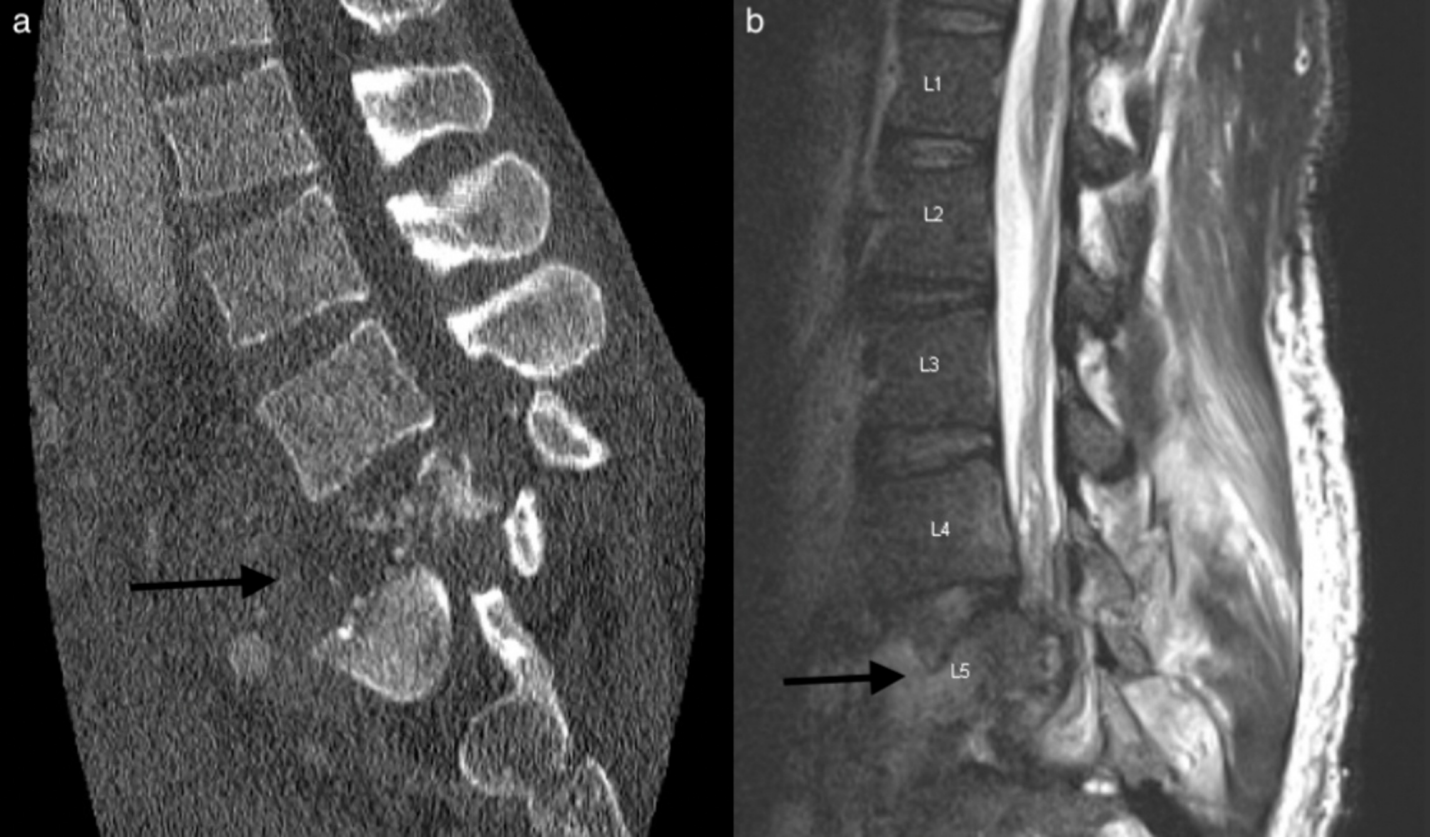
Preoperative CT and MRI of lumbar spine (a) CT lumbar spine in the sagittal plan demonstrating L5 burst fracture. (b) T2 lumbar MRI in the sagittal plane demonstrating severe destructive process of L5 with extensive epidural and soft tissue enhancement concerning for infection.

A male patient in his 30s presented with a three-month history of progressively worsening lower back pain, bilateral lower extremity radiculopathy, and bilateral lower extremity edema. He reported feeling a pop in his back when he fell while lifting a heavy object around the time of the onset of symptoms but denied any major trauma. He denied any associated fever, sensory changes, strength deficits, or bowel or bladder incontinence. He was diagnosed with a compression fracture at an outside hospital at the time of the incident. When he presented to our institution, a CT scan demonstrated a three-column fracture dislocation of L5 with grade 4 retrolisthesis of L4 on L5, and MRI revealed severe stenosis with extensive epidural and soft tissue enhancement concerning for infection (Figure 1). The suspected infection appeared to be encasing the IVC and iliac veins, consistent with the patient’s edema.

The patient noted a remote history of intravenous drug use but denied use for several years. He had an unremarkable medical history aside from chronic hepatitis C. Labs on admission were significant for mildly elevated white blood cell (WBC) count of 11.32 x 10^9^/L, elevated erythrocyte sedimentation rate (ESR) of 120 mm/hr, and elevated C-reactive protein (CRP) of 7.5 mg/L. No preoperative antibiotics were given.

An anterior corpectomy with cage placement to be followed by a stage II posterior fusion with pedicle screws from L3 to S1/ilium was planned. Vascular surgery was asked to help with the anterior approach in order to safely dissect inflammatory tissue off of the major veins. However, extensive adhesions and poor dissection planes led to the anterior approach being aborted. A lateral approach was considered, but review of imaging demonstrated unfavorable orientation of the ilium.

**Figure 2:**
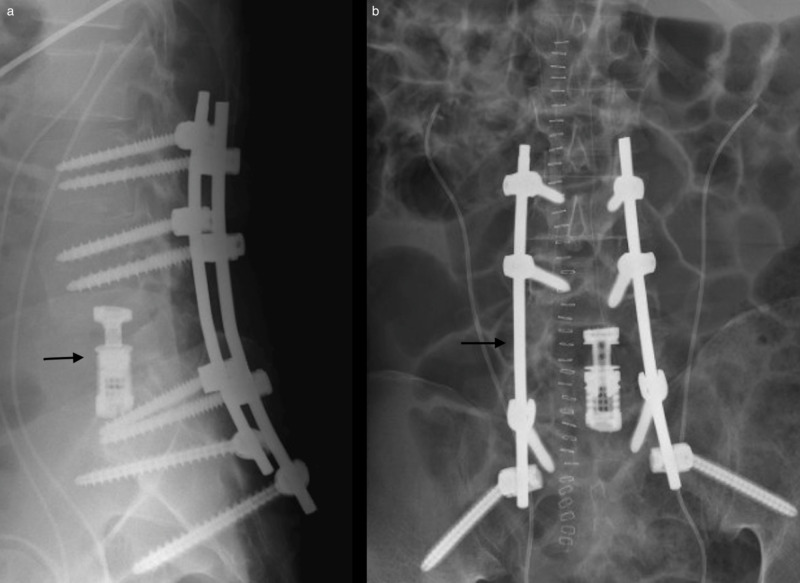
X-rays of postoperative hardware (a) Lateral x-ray demonstrating fused interbody cage between L4 and S1. (b) Posteroanterior x-rays demonstrating postoperative hardware with rods, bilateral sacroiliac screws, and pedicle screws extending from L3 to S1.

Thus, a posterior corpectomy with placement of an expandable cage to be followed by pedicle screw placement from L3-S1/ilium was planned. Intraoperatively, extensive fibrinous epidural tissue and purulence were encountered. Instrumentation from L3 to the ilium was placed, skipping L5. A complete L5 bilateral pediculectomy was performed with a high-speed drill, curettes, and rongeurs. A left-sided temporary rod was placed. A right-sided transpedicular approach was then utilized for the posterior L5 corpectomy. This side was selected given that the majority of the osseous destruction and retropulsed fragments were eccentric to the right. Of note, thick fibrinous, inflammatory tissue was encountered in the L5 body along with significant liquid pus. Samples of tissue were taken for histopathological analysis and for cultures. Pituitary rongeurs, Kerrison dissectors, and a drill were utilized to complete the corpectomy. We then proceeded with placement of our interbody cage. The right L4 nerve root was retracted superiorly, and the L5 root was retracted inferiorly to provide adequate space for cage placement. The interbody cage was cautiously inserted horizontally in between these roots and then rotated vertically to begin meeting the endplates. Our temporary rod was loosened prior to expansion of the cage. The cage was successfully expanded and had secure purchase against both endplates. The final rods were then placed (Figure 2). The patient did well postoperatively with complete resolution of his preoperative lower extremity edema and significant improvement in his radicular pain.

**Figure 3:**
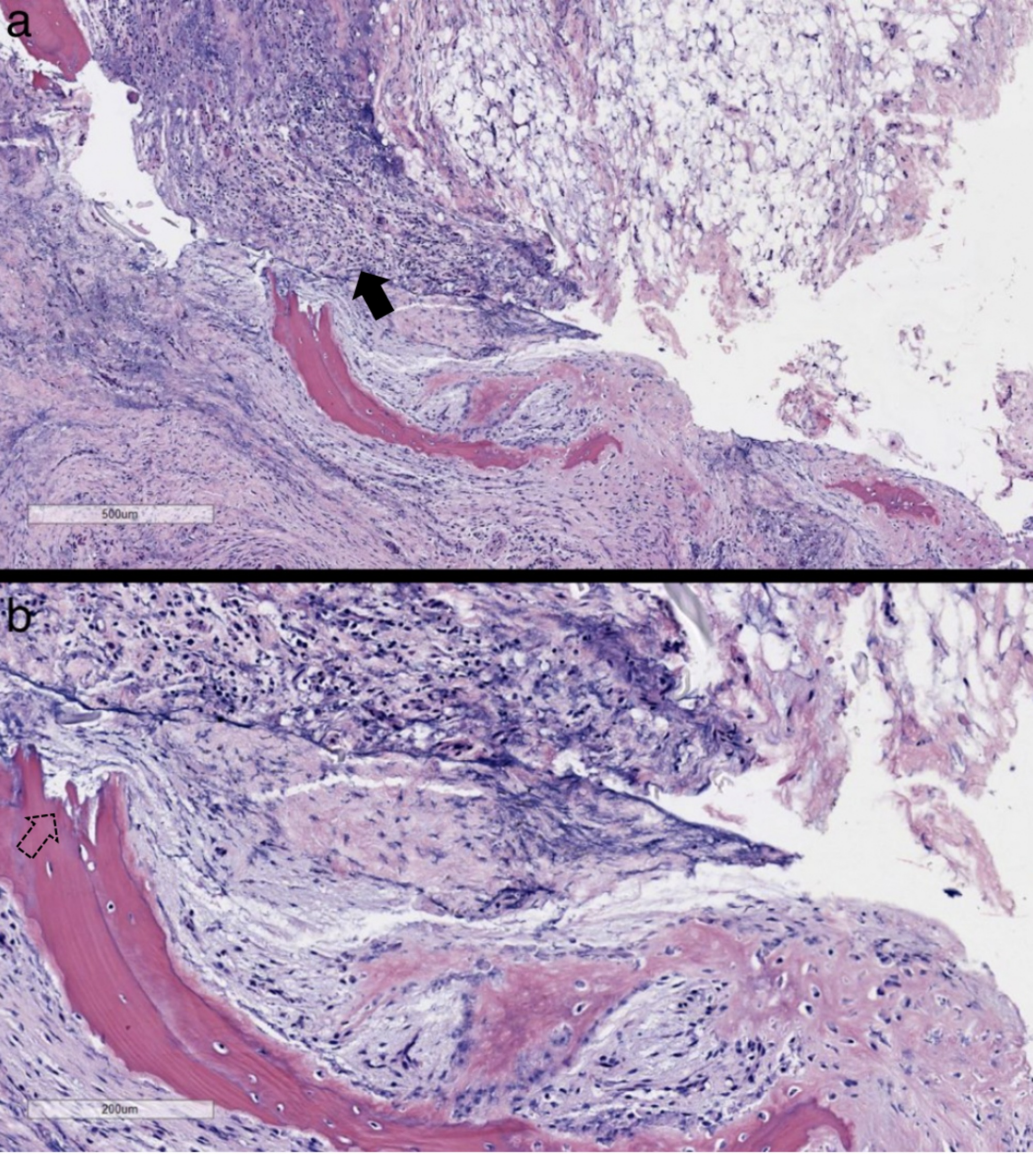
Biopsy of epidural phlegmon with bone fragments Hematoxylin and eosin stain (H&E) at ×4 (a) and ×10 (b). These representative images of the H&E slides reveal acute and chronic inflammation (solid arrow) in the soft tissue, fibrosis, and granulation tissue, with bone fragments indicative of bone destruction and repair (dashed arrow). Although similar to an abscess, a phlegmon is unbound and can spread along connective tissue planes and muscle fibers. Ziehl-Neelsen and Grocott’s methenamine silver stains are negative for organisms.

Histopathological analysis demonstrated acute and chronic inflammation with granulation tissue (Figure 3). Bone tissue Mycobacterium tuberculosis DNA polymerase chain reaction, urine Histoplasma antigen, and blood Brucella antibody tests were negative. Final aerobic, anaerobic, fungal, and mycoplasma cultures were also negative. Gram stains, Ziehl-Neelsen stains, and methenamine silver stains failed to identify any organisms.

He was discharged on postoperative day 5 with follow-up scheduled for two weeks later. At the time of discharge, he was ambulating well without difficulty. Follow-up labs inflammatory markers were collected three months following the procedure, which were significant for improved CRP of 1.0 mg/L and ESR of 54 mm/hr.

## Discussion

The preceding case described an adult male who successfully underwent an uncomplicated posterior L5 corpectomy with placement of an expandable cage for a suspected cold abscess that presented as a three-column fracture.

Overall, there is a paucity of literature that addresses posterior corpectomy in the lumbar spine with expandable cage placement, particularly at the L5 level. This is likely due to a combination of factors such as rarity of an L5 burst fracture or three-column fracture and the relative safety of an anterior L5 approach [19]. With a posterior approach, there is a risk of lumbar nerve root damage, which would result in motor deficits. Furthermore, matching the endplates and achieving the necessary lordosis at the lumbosacral junction can add an additional element of complexity. We initially attempted an anterior approach with dissection of inflammatory tissue off of the IVC and iliac veins, but this was aborted due to tissue adhesion and a high risk of iatrogenic damage. Limited by the patient’s anatomy, we instead performed a posterior L5 corpectomy.

This case demonstrated the ability of spinal infections in the lumbar spine to involve the IVC and iliac vessels. Surgeons should be aware of lower extremity edema when assessing for possible lumbar spinal infection, as involvement of these vessels could be life threatening. Additionally, there is novelty in that this patient presented with a nontraumatic fracture involving all three columns of L5 and a grade 4 retrolisthesis in the setting of a cold abscess. A review of literature did not reveal this degree of deformity and instability due to a subacute infection.

The posterior-only approach for L5 corpectomy with expandable cage placement was found in literature to be performed in the setting of metastasis, trauma, and infection [15-18]. Hunt et al. described the transpedicular approach in the setting of a three-column metastasis of L5 [17]. Kocis et al. described the posterior approach in the setting of a traumatic complete burst fracture dislocation with disruption of the posterior ligament [18]. Lu et al. described one case of posterior corpectomy with expendable cage placement, with no complications and resolution of symptoms, at the L4-L5 level in the setting of osteomyelitis [20]. To our knowledge, this is the first description of a three-column L5 fracture dislocation with severe listhesis in the setting of a subacute infection with cold abscess. The large amount of inflammatory tissue added to the complexity of the case by requiring careful dissection of tissue off of vital structures. This case demonstrates that the posterior-only approach can be considered in the setting of L5 fracture dislocation with severe deformity in cases where the other approaches are not viable.

There are, however, some important limitations to note. This was a case report on a single patient. Larger retrospective studies would be necessary to at least determine complication rates. Prospective studies are necessary to compare efficacy. However, given the rarity of L5 pathology necessitating such a procedure and the importance of proper patient selection, a randomized, controlled trial may not be feasible.

## Conclusions

Infectious processes of the lumbar spine have proven to present in various ways including vertebral fractures, local abscesses, and infiltration and compression of local structures. Cases with significant instability or neurological compromise require surgical intervention. For patients with three-column fractures of L5 due to an inflammatory process or trauma, a single-stage posterior corpectomy with placement of an expandable cage may be considered as an appropriate treatment option.
